# Cassava by-products in broiler diets: effects on growth performance based on a systematic review

**DOI:** 10.1007/s11250-026-05289-y

**Published:** 2026-08-12

**Authors:** Wanessa Daróz Matte, Cynthia Pieri Zeferino, Henrique Melo da Silva, Jean Kaique Valentim, Melody Martins Cavalcante Pereira, Maria Eduarda Vignoli Nabhan, Lívia Costa Silva, Sarah Sgavioli

**Affiliations:** 1 ¹Postgraduate Program in Animal Production, Universidade Brasil, Descalvado, São Paulo, 13690-000 Brazil; 2 Dairy Cattle Research Lab, Universidade Federal de Mato Grosso, Sinop, Mato Grosso 78557-267 Brazil; 3 Department of Animal Production, Universidade Federal Rural do Rio de Janeiro, Seropédica, Rio de Janeiro, 23890-000 Brazil; 4 Graduate Program in Animal Science, Pontifícia Universidade Católica do Paraná, Curitiba, Paraná 80215-901 Brazil

**Keywords:** Alternative feed ingredients, Broiler chickens, Cassava by-products, Corn replacement, Feed efficiency, Growth performance, Systematic review

## Abstract

The use of cassava (*Manihot esculenta* Crantz) by-products as alternative energy sources in broiler diets has increased, but their effects on growth performance remain inconsistent across studies. This systematic review evaluated the effects of replacing corn with cassava by-products (cassava meal, pulp, and bagasse) on broiler performance using a qualitative direction-of-effect synthesis. A total of 36 studies published between 2005 and 2025 were included, comprising 187 dietary comparisons. Most studies were conducted in Brazil (36.1%), Nigeria (25.0%), and Indonesia (18.2%). Because measures of variability were frequently unavailable, responses in feed intake (FI), body weight gain (BWG), and feed conversion ratio (FCR) were classified as positive, negative, or neutral relative to the reference treatment. Feed intake was predominantly unaffected (54.4% neutral responses). For BWG, negative responses were most frequent (49.2%), followed by neutral (44.4%) and positive responses (6.4%). For FCR, neutral (45.5%) and negative (44.4%) responses occurred at similar frequencies, whereas positive responses accounted for 10.2% of comparisons. Negative responses became more frequent as dietary inclusion levels increased, particularly for BWG and FCR, and were most evident at inclusion levels above 25%. Most studies (86.1%) were classified as having a high risk of bias or some concerns, and measures of variability were frequently not reported, limiting quantitative comparison of effect estimates and the strength of inference. Overall, cassava by-products were more frequently associated with reduced growth performance at higher dietary inclusion levels, although responses varied according to by-product type, processing method, inclusion level, and study conditions. These findings support cautious dietary formulation and highlight the need for standardized experimental reporting to better define safe and effective inclusion levels for individual cassava by-products in broiler nutrition.

## Introduction

In traditional poultry production systems, corn and soybean meal account for approximately 90% of the nutritional composition of diets. However, in least-cost feed formulations, alternative ingredients may be incorporated to optimize production costs depending on their local availability (Uchegbu et al. 2011). Among these alternatives, cassava (Manihot esculenta Crantz) is particularly viable in tropical countries because of its high starch content (Latif and Müller, 2015) and broad availability. Brazil produced 20.57 million tons of cassava harvested from 1.3 million hectares as of September 2025 (IBGE, 2025).

Starch-processing industries generate large quantities of by-products, such as bagasse and bran, during cassava industrialization. These by-products consist largely of fibrous root material containing residual starch that is not extracted during processing (Leonel and Cereda 2000). Driven by sustainability goals, there is increasing interest in incorporating these by-products into animal nutrition, particularly due to their reduced environmental contamination associated with hydrogen cyanide (HCN) (Cereda 2001; Nambisan 2011; Oliveira et al. 2012). The main limitations to the use of cassava in poultry diets are its high crude fiber content, the presence of HCN, and its low crude protein concentration (Diarra and Devi 2015).

The metabolizable energy value of cassava residues is approximately 2973 kcal ME/kg, making them a feasible substitute for corn in poultry feeding programs (Rostagno et al. 2024). The solid residue resulting from cassava starch extraction contains fibrous material (approximately 11% crude fiber) and non-extracted starch (60–70%), with minimal amounts of crude protein, lipids, vitamins, and minerals (Aro et al. 2010). Nutrient composition varies according to cassava cultivar, plant maturity, and processing method, among other factors (Pandey et al. 2000), and these factors must be considered and properly adjusted during diet formulation.

According to Ogbuewu and Mbajiorgu (2023), up to 25% cassava can be included in layer diets without adverse effects on feed intake, feed conversion ratio, or egg production, indicating a promising future for cassava as an energy source for laying hens. However, although scientific literature on cassava by-products in broiler nutrition exists, the available evidence remains fragmented, with considerable variation in by-product type, dietary inclusion level, rearing phase, and reported performance outcomes. Consequently, a systematic review using a qualitative direction-of-effect synthesis was conducted to identify overall response patterns and knowledge gaps in the current literature. The objective of the present systematic review was to evaluate the effects of replacing corn with cassava by-products in broiler diets on growth performance across different rearing phases using a qualitative direction-of-effect synthesis.

## Materials and methods

### Literature search strategy

A systematic literature search was conducted in the databases Web of Science, Scopus, and PubMed to identify studies evaluating the use of cassava by-products in broiler diets. The search was performed in March 2025 and included studies published between 2005 and 2025. The search strategy was developed based on a Population–Intervention–Outcome (PIO) framework, where Population was defined as broiler chickens, Intervention as dietary inclusion of cassava by-products, and Outcome as growth performance parameters. This systematic review was conducted according to the PRISMA guidelines.

The following search string was used: (cassava OR “cassava by-product*” OR “cassava residue*” OR “cassava pulp” OR “cassava flour” OR “cassava bagasse”) AND (broiler* OR “broiler chicken*” OR poultry) AND (performance OR “growth performance” OR “body weight gain” OR “feed intake” OR “feed conversion ratio”) AND (diet* OR feed OR nutrition OR “corn replacement”). Search terms were adapted as needed for each database. In addition, the reference lists of all eligible studies were manually screened to identify potentially relevant articles not retrieved through the electronic database search. Only studies published in English in peer-reviewed journals were considered. This language restriction may have introduced language bias and should be considered when interpreting the findings. This systematic review was not prospectively registered in an international review registry.

### Study selection and eligibility criteria

All retrieved records were imported into Rayyan for screening and duplicate removal (Ouzzani et al. 2016). Two independent reviewers screened titles and abstracts, followed by full-text assessment. Disagreements were resolved through discussion or consultation with a third reviewer.

Studies were included if they met the following criteria:

1. In vivo experimental studies involving broiler chickens;
2. Evaluation of diets containing cassava by-products (pulp, bagasse, or meal);
3. Inclusion of a control or reference group;
4. Reporting at least one performance outcome (feed intake, body weight gain, or feed conversion ratio);
5. Cassava by-products used as partial or total replacement of corn without confounding inclusion of other major energy sources.

Studies were excluded if they: (i) involved other poultry species; (ii) did not report performance outcomes; (iii) included cassava in combination with other major dietary energy replacements that prevented isolated evaluation; (iv) were not published in English.

### Data extraction and database construction

Data were extracted independently by two reviewers using a standardized spreadsheet. Extracted information included: study identification (authors, year, country); experimental design (number of animals, replicates, duration); type of cassava by-product (pulp, bagasse, meal); dietary inclusion level (%); production phase (starter, grower–finisher, total period); performance outcomes (FI, BWG, FCR); nutritional characteristics of diets when available. When discrepancies occurred, consensus was reached through discussion. Each dietary treatment within a study was recorded as a separate comparison for qualitative synthesis.

### Risk of bias assessment

Risk of bias was assessed using the Cochrane Collaboration Risk of Bias 2 (RoB 2) tool (Sterne et al. 2019), adapted for animal nutrition studies according to the recommendations of Sargeant and O’Connor (2014), as no universally accepted risk-of-bias assessment tool is currently available for controlled animal nutrition experiments. Five domains were evaluated: D1: randomization process; D2: deviations from intended interventions; D3: missing outcome data; D4: measurement of outcomes; D5: selection of reported results.

Each domain was classified as low risk, some concerns, or high risk. Assessments were conducted independently by two reviewers, with disagreements resolved by consensus. Results were visualized using the “robvis” package (McGuinness & Higgins 2021) in R.

### Data synthesis and direction-of-effect analysis

A qualitative synthesis based on a direction-of-effect approach was conducted to summarize the effects of cassava by-products on broiler growth performance. This approach was selected because many of the included studies did not consistently report measures of variability (e.g., standard deviation or standard error), precluding quantitative comparison of effect estimates across studies.

Each comparison was classified as positive (+), negative (−), or neutral (0) relative to the control group (0% inclusion), or to the lowest inclusion level when a control group was not available. For feed intake (FI) and body weight gain (BWG), increases relative to the reference were classified as positive and decreases as negative. For feed conversion ratio (FCR), reductions (improved efficiency) were considered positive, whereas increases were considered negative.

Classification was based on reported mean values regardless of statistical significance because variability measures were inconsistently reported across studies. When regression models were presented, classification was based on the biological response pattern.

Quadratic responses were considered positive when performance improved up to an apparent optimal inclusion level. Each dietary inclusion level within a study was treated as a separate comparison for descriptive purposes; however, this approach may introduce dependency among observations and is acknowledged as a limitation of the qualitative synthesis. To facilitate interpretation, inclusion levels were grouped into predefined categories low (2–8%), moderate (10–25%), and high (> 25%)), allowing identification of overall response patterns across studies.

## Results

### Study selection and characteristics

**Fig. 1:**
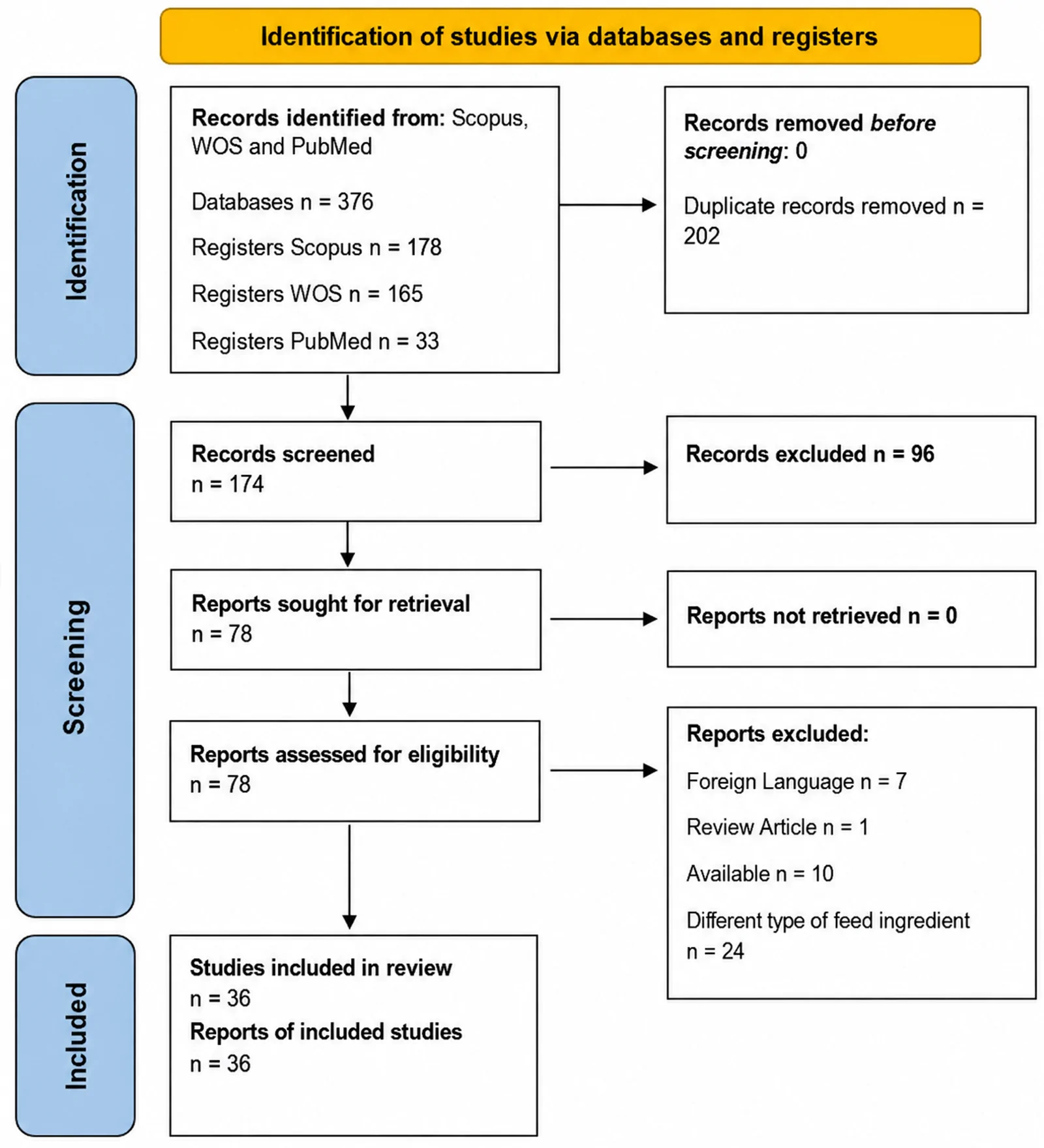
PRISMA flow diagram of the study selection process for the systematic review

The literature search identified 376 records, of which 202 duplicates were removed. After title and abstract screening, 96 studies were excluded for not meeting the eligibility criteria, resulting in 78 articles for full-text assessment. Of these, 42 were excluded due to absence of relevant performance outcomes, inclusion of confounding dietary ingredients, or language restrictions. A total of 36 studies met the eligibility criteria and were included in the qualitative synthesis (Fig. 1). The included studies were published between 2005 and 2025 and were predominantly conducted in Brazil (36.1%), Nigeria (25.0%), and Indonesia (18.2%). Cassava pulp was evaluated in 24 studies (66.7%), followed by cassava flour in seven studies (19.4%) and cassava bagasse in five studies (13.9%) (Table 1).

**Table 1:** Characteristics of the studies included in the systematic review, including author, year of publication, country, cassava by-product, production phase, and performance outcomes evaluated

Code	Author	Year	Country	By-product	Phases	Performance*
1	Do Nascimento et al.	2005	Brazil	Pulp	Grower/Finisher	FI, BWG, FCR
2	Khempaka et al.	2009	Thailand	Pulp	Grower/Finisher	FI, BWG, FCR
3	Aderemi et al.	2010	Nigeria	Flour	Starter and Grower/Finisher	FI, BWG, FCR
4	Ali-Mursyid et al.	2010	Indonesia	Bagasse	Total	FI, BWG, FCR
5	Ferreira et al.	2012	Brazil	Pulp	Grower/Finisher	FI, BWG, FCR
6	Sousa et al.	2012	Brazil	Bagasse	Starter and Grower/Finisher	FI, BWG, FCR
7	Tang et al.	2012	Australia	Pulp	Starter; Grower/Finisher and Total	FI, BWG, FCR
8	Oso et al.	2014	Nigeria	Pulp	Total	FI, BWG, FCR
9	Tesfaye et al.	2013	Ethiopia	Pulp	Starter and Finisher	FI, BWG, FCR
10	Akapo et al.	2014	Nigeria	Pulp	Total	FI, BWG, FCR
11	Khempaka et al.	2014	Japan	Pulp	Total	FI, BWG, FCR
12	Lubis et al.	2007	Indonesia	Bagasse	Starter and Grower/Finisher	FI, BWG, FCR
13	Oladunjoye et al.	2014	Nigeria	Flour	Total	FI, BWG, FCR
14	Picoli et al.	2014	Brazil	Pulp	Grower/Finisher	FI, BWG, FCR
15	Geron et al.	2015	Brazil	Pulp	Total	FI, BWG, FCR
16	Holanda et al.	2015	Brazil	Flour	Grower/Finisher	FI, BWG, FCR
17	Sugiharto et al.	2016	Indonesia	Pulp	Grower/Finisher	BWG, FCR
18	Broch et al.	2017	Brazil	Bagasse	Starter and Total	FI, BWG, FCR
19	Ojewola et al.	2017	Nigeria	Flour	Total	FI, BWG, FCR
20	Dayal et al.	2018	Nigeria	Flour	Grower and Grower/Finisher	FI, BWG, FCR
21	Parente et al.	2018	Brazil	Bagasse	Grower/Finisher	FI, BWG, FCR
22	Da Silva et al.	2019	Brazil	Pulp	Grower/Finisher	FI, BWG, FCR
23	Sugiharto et al.	2019	Indonesia	Pulp	Grower/Finisher	FI, BWG, FCR
24	Yadav et al.	2019	United States (Hawaii)	Pulp	Starter and Grower/Finisher	FI, BWG, FCR
25	Chang’a et al.	2019	Australia	Pulp	Starter; Grower/Finisher and Total	FI, BWG, FCR
26	Oyewole et al.	2020	Nigeria	Pulp	Total	FI, BWG, FCR
27	Trautenmüller et al.	2020	Brazil	Pulp	Starter; Grower/Finisher and Total	FI, BWG, FCR
**28**	**Almeida et al.**	2020	**Brazil**	**Pulp**	**Starter; Grower/Finisher and Total**	**FI**,** BWG**,** FCR**
29	Ferreira et al.	2021	Brazil	Pulp	Starter and Grower/Finisher	FI, BWG, FCR
30	Ojediran et al.	2022	Nigeria	Pulp	Total	FI, BWG, FCR
31	Okpara et al.	2022	Nigeria	Pulp	Total	FI, BWG, FCR
32	Vieira et al.	2022	Brazil	Flour	Grower/Finisher	FI, BWG, FCR
33	Puspita et al.	2023	Indonesia	Pulp	Starter and Grower/Finisher	FI, BWG, FCR
34	Ramadhani et al.	2025	Indonesia	Pulp	Grower/Finisher	FI, BWG, FCR
35	Yang et al.	2024	China	Pulp	Starter and Grower/Finisher	FI, BWG, FCR
36	Osei-Adjei et al.	2025	Ghana	Flour	Grower/Finisher	FI, BWG, FCR

FI: feed intake; BWG: body weight gain; FCR: feed conversion ratio.

Most studies evaluated the grower–finisher phase (33.3%) or the total rearing period (27.8%), with fewer studies focusing exclusively on the starter phase or combined production phases. Feed intake (FI), body weight gain (BWG), and feed conversion ratio (FCR) were reported in 97.2% of the included studies (Table 1).

### Risk of bias assessment

**Fig. 2:**
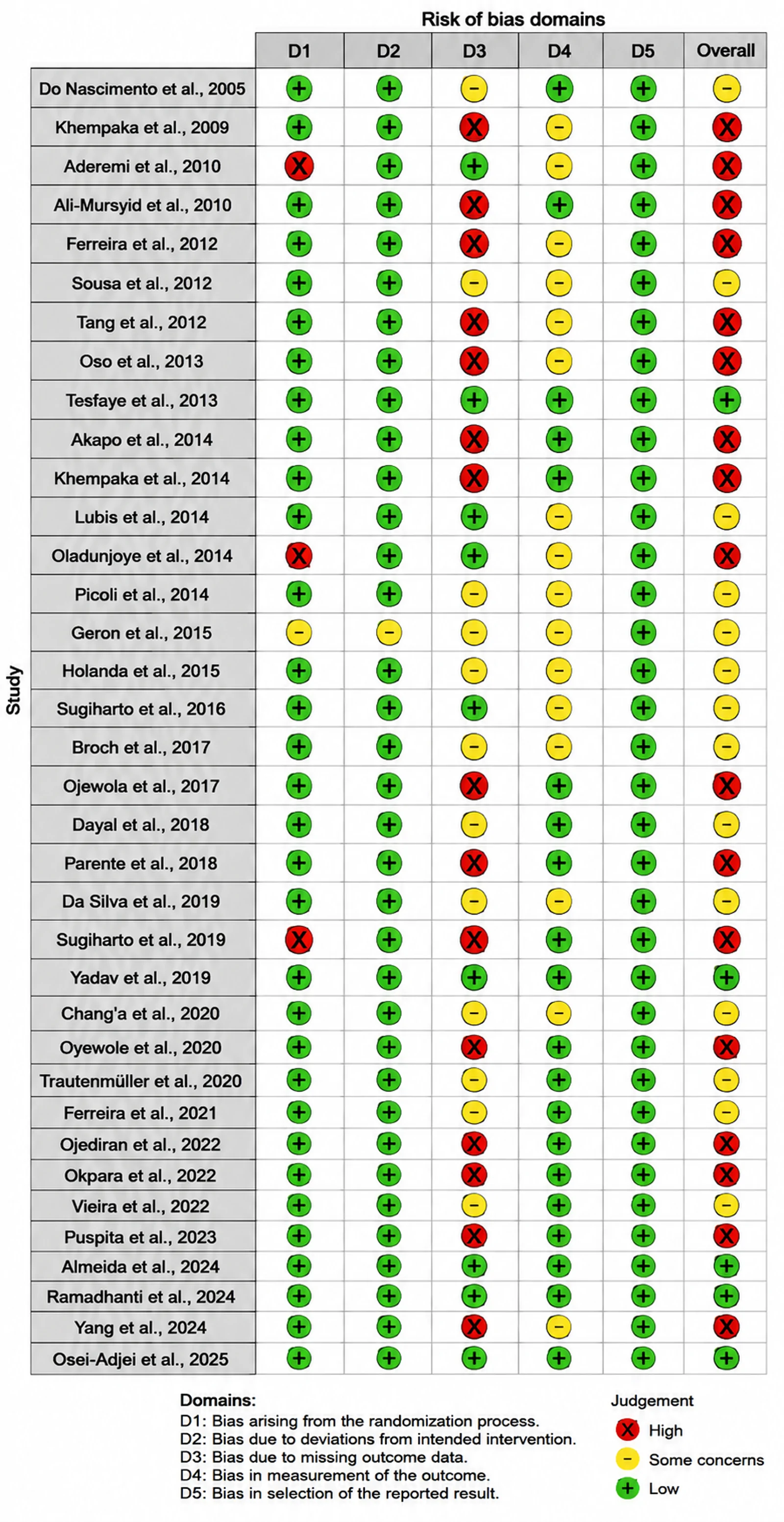
Risk-of-bias assessment of the included studies across the five evaluated domains using the adapted RoB 2 tool

Overall, 13.9% of the studies were classified as having low risk of bias, whereas 47.2% were classified as high risk and 38.9% as presenting some concerns. The most frequent sources of bias were related to missing outcome data (Domain 3) and measurement of outcomes (Domain 4) (Fig. 2). Domains related to deviations from intended interventions (Domain 2) and selection of reported results (Domain 5) showed the highest proportion of low-risk classifications.

### Direction-of-effect synthesis

**Fig. 3:**
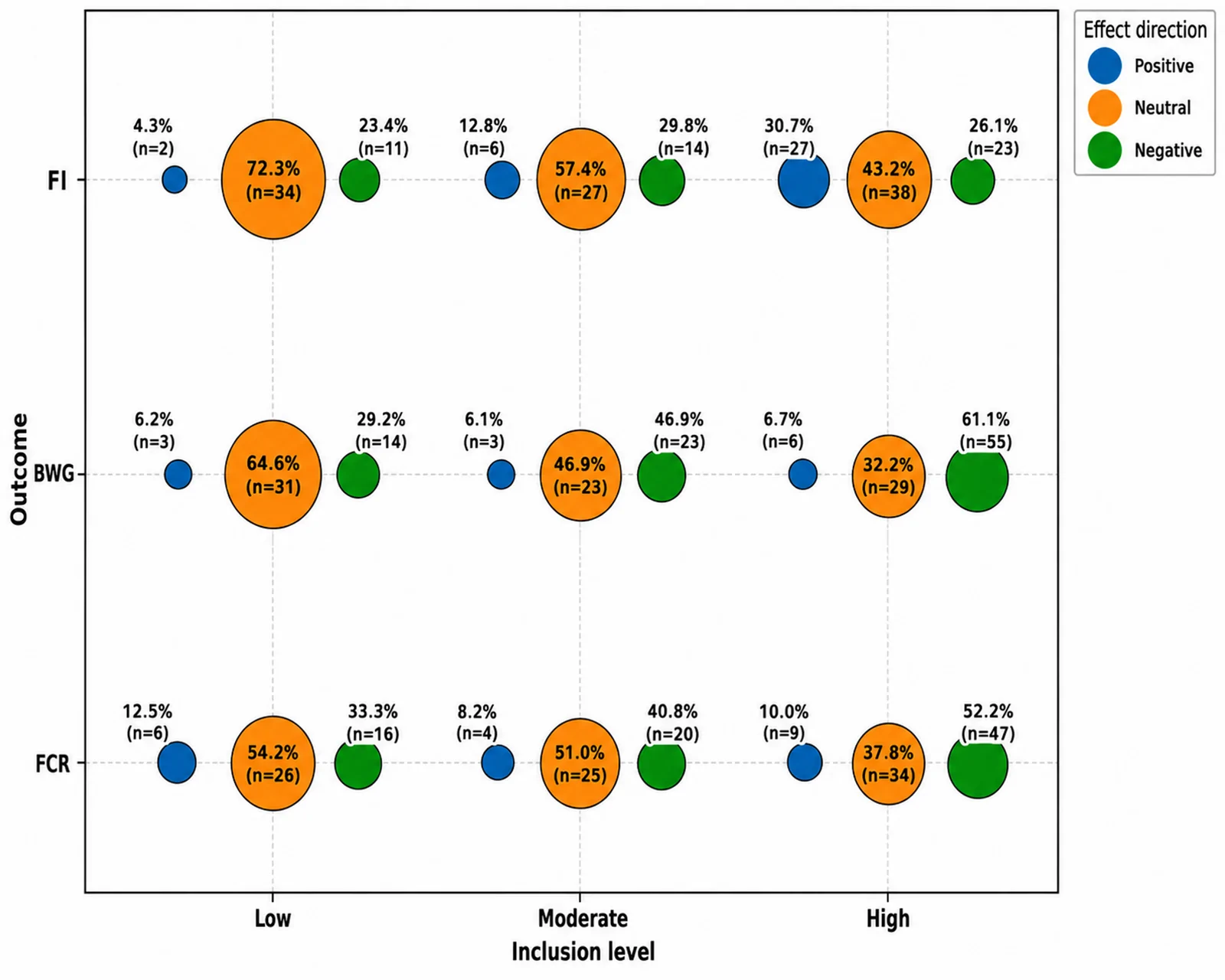
Direction-of-effect responses for feed intake (FI), body weight gain (BWG), and feed conversion ratio (FCR) according to dietary inclusion level (%) in broilers fed cassava by-products

A total of 187 comparisons were included in the qualitative synthesis. For feed intake (FI), neutral responses were the most frequent (54.4%), followed by negative (26.4%) and positive responses (19.2%). For body weight gain (BWG), negative responses were the most frequent (49.2%), followed by neutral (44.4%) and positive responses (6.4%). For feed conversion ratio (FCR), neutral (45.5%) and negative (44.4%) responses occurred at similar frequencies, whereas positive responses accounted for 10.2% of the comparisons (Fig. 3).

### Influence of inclusion level

When comparisons were stratified according to dietary inclusion level (2–8%, 10–25%, and > 25%), negative responses became more frequent with increasing inclusion level, particularly for body weight gain (BWG) and feed conversion ratio (FCR) (Fig. 3). In contrast, neutral responses were more frequent at lower dietary inclusion levels for all evaluated outcomes. Feed intake (FI) responses were distributed across all inclusion categories, with neutral responses remaining the most frequent regardless of dietary inclusion level (Fig. 3).

### Relationship between risk of bias and direction of effect

Most comparisons were derived from studies classified as having high risk of bias or some concerns. No clear relationship was observed between the risk-of-bias category and the direction of effect across the evaluated outcomes.

## Discussion

The present systematic review provides a comprehensive synthesis of the available evidence on the use of cassava by-products as partial or total replacements for corn in broiler diets. The results indicate that these ingredients were more frequently associated with reduced growth performance, particularly at dietary inclusion levels above 25%, although considerable variability was observed among studies.

A plausible explanation for these findings is the high fiber concentration present in cassava by-products. With increased inclusion in the diet, the amount of carbohydrates with glycosidic bonds (α-1,6 β-1,4, and β-1,6), which are resistant to hydrolysis in the digestive tract, also increases, potentially affecting the digestion of other nutrients, leading to more significant nutrient loss (Raupp et al. 2002) and reduced nutrient availability.

Physiologically, birds have limited utilization of fibrous compounds due to their digestive system characteristics and nutritional interactions, which affect nutrient digestion (Costa et al. 2009; Macari and Maiorka 2017).

Additionally, the high fiber content in food may increase digesta viscosity in the chicken small intestine, decreasing the digestibility and absorption of other nutrients (Hetland et al. 2004). When this fiber-rich content comes into contact with water, gums are formed that increase bolus size, and through gastric distension, FI is reduced due to a feeling of satiety (Macari and Maiorka 2017). This physiological mechanism explains why fiber-rich foods contribute to reduced FI in chickens.

The low protein content of cassava by-products, along with the challenges of high fiber content and anti-nutritional factors, limits their inclusion (Valdivié et al. 2008). Consequently, one key to the successful use of cassava by-products as a corn substitute in poultry diets is determining the appropriate replacement level and product origin to ensure adequate nutritional composition concerning lysine and methionine (Okereke 2012).

Moderate levels of dietary fiber have been shown to improve nutrient digestibility and performance in poultry, primarily due to their role in the development of the gastrointestinal tract and in modulating intestinal content characteristics (Röhe and Zentek 2021). In addition, studies indicate that supplementation with soluble or insoluble fiber can be beneficial to poultry health, with recommended inclusion levels ranging from 2 to 2.4% in the diet (Jha and Mishra 2021).

Despite the recognized importance of fiber for intestinal histomorphology and nutrient digestibility, only 28% of the studies included in the present review (10 out of 36) evaluated these parameters.

Measures of variability, such as standard deviation (SD) or standard error of the mean (SEM), were not consistently reported across the included studies, preventing reliable quantitative comparison of effect estimates. Consequently, the evidence was synthesized using a qualitative direction-of-effect approach (Higgins and Green, 2023).

The omission of SD was the most prominent issue, affecting 85.7% (30 of 35 studies) for feed intake and 86.1% (31 of 36 studies) for both feed conversion ratio and body weight gain. The absence of SEM was slightly lower, occurring in 42.9% (15 of 35 studies) for feed intake and in 38.9% (14 of 36 studies) for feed conversion ratio and weight gain. These omissions limited the assessment of estimate precision and within-study variability.

The direction-of-effect assessment showed a predominance of neutral responses for feed intake (FI), whereas negative responses were more frequently observed for body weight gain (BWG) and, to a lesser extent, feed conversion ratio (FCR). These findings suggest that cassava by-products were more frequently associated with changes in growth performance than with voluntary feed intake.

In broiler chickens, feed intake is primarily regulated by dietary energy density; thus, birds tend to increase intake to compensate for diets with lower metabolizable energy (Macari and Maiorka 2017). However, this compensatory response may be constrained by physical and physiological factors, such as increased dietary fiber, gut fill, and digesta viscosity, which can limit intake capacity (Rostagno et al. 2024).

As a result, even when feed intake remains unchanged, reduced nutrient digestibility and energy availability may impair growth, leading to lower BWG and poorer FCR. The greater frequency of neutral responses for FI compared with BWG and FCR suggests that growth performance was more sensitive to increasing dietary inclusion levels of cassava by-products than voluntary feed intake.

These findings should be interpreted considering the methodological limitations and overall quality of the included studies.

In a meta-regression conducted by Ogbuewu and Mbajiorgu (2023), evaluating cassava inclusion in diets for laying hens, the authors observed that substitution levels above 25% increased feed intake and reduced eggshell thickness, egg production, and egg weight. These adverse effects may be associated with antinutritional factors (Omede et al. 2017) found in cassava-based diets that exceeded the tolerance threshold of laying hens (Chauynarong et al. 2009). Similarly, the wide variation in dietary inclusion levels observed in the present review may have contributed to the heterogeneous performance responses reported among studies. Dietary inclusion levels ranged from 2 to 100% for cassava pulp, 2.5 to 30% for cassava bagasse, and 6.8 to 100% for cassava meal, highlighting the lack of standardization across experiments.

Cassava pulp. Cassava pulp is the by-product with the least industrial processing, resulting in minimal alteration to its nutritional composition and a higher fiber concentration than other cassava by-products. The pulp typically contains 50–70% starch and 20–30% fiber, mainly cellulose and nonstarch polysaccharides (Rattanachomsri et al. 2009).

Cassava bagasse. Cassava bagasse is a by-product of starch extraction (Leonel and Cereda 2000). During processing, cassava roots are peeled, crushed, and washed to extract starch, resulting in a coarse residue. This material typically contains approximately 1.5% crude protein, 11% crude fiber, and 2,450 kcal/kg of metabolizable energy on a dry matter basis (Rodrigues and Campos 2001), and may contain up to 80% residual starch.

Cassava meal. According to Rostagno et al. (2024), whole cassava root meal contains 2.64% crude protein, 4.21% crude fiber, 73.7% starch, and 2,973 kcal/kg of metabolizable energy for poultry.

An economic analysis by Sousa et al. (2012) examined the inclusion of cassava bagasse in two rearing phases (from d 1–21 and d 22–40) in broiler chicken production. The results indicated that the lowest feed cost per kilogram of live weight gain and the best productive efficiency index were achieved with dietary inclusion levels of up to 5% cassava bagasse, indicating increased profitability. Above 5%, feed costs increased because of the need to supplement amino acids to maintain a balanced diet.

In regions with high cassava production, safe levels of corn substitution that do not impair broiler performance could promote sustainable production by utilizing these by-products and reducing diet costs (Uchegbu et al. 2011). Cassava by-products can be included in monogastric diets as a strategy to reduce corn use and, consequently, feed costs (Ferreira et al. 2012). However, their main limitation is the high fiber content, which often reduces nutrient utilization. High levels of crude fiber, particularly from plant cell wall components, can negatively affect diet digestibility (Brito et al., 2008).

The risk-of-bias assessment showed that only 13.9% of the studies were classified as having a low risk of bias (Tesfaye et al. 2013; Yadav et al. 2019; Almeida et al., 2020; Ramadhanti et al., 2025; Osei-Adjei et al. 2025), whereas the remaining studies were classified as presenting either some concerns or a high risk of bias. Missing outcome data were identified in 15 studies (41.6%), mainly due to bird mortality, without appropriate methodological adjustments to account for its effects on feed intake and feed conversion ratio. In addition, 16 studies (44.4%) presented subjective methodologies or lacked sufficient detail regarding the measured variables, likely due to the assumption of standard practices in performance evaluation.

Overall, 86.1% of the studies were classified as having high risk of bias or some concerns, indicating important methodological limitations across the evidence base. Although these studies provide useful quantitative data, their results should be interpreted with caution, and improvements in experimental design and reporting are needed in future research.

The geographic distribution of the included studies showed that Brazil, Nigeria, and Indonesia accounted for the majority of studies, with the cassava species *Manihot esculenta* being the most commonly used across these regions.

The processing methods of cassava by-products varied widely among studies. Most involved dehydration, either sun-drying or industrial drying, while others included grinding, washing, peeling, and, in fewer cases, fermentation. In general, studies used residues from pulp or bagasse processing with relatively simple processing methods, regardless of geographic origin. A temporal analysis indicated a progression from traditional sun-drying and simple grinding techniques to more advanced methods, such as oven-drying, evaluation of cyanogenic compound volatilization, and controlled fermentation using microorganisms, which may contribute to improved utilization of cassava by-products.

 Regarding hydrogen cyanide (HCN) content, only seven studies reported its concentration, with values ranging from 3.97 to 43.40 mg/kg. Cassava is considered one of the most important cyanogenic species in tropical regions due to the natural presence of linamarin and lotaustralin (Cereda and Mattos 1996). Other economically important plants, such as sorghum, plum, bamboo, and rubber tree, also contain varying levels of HCN (Haque and Bradbury 2002), which can lead to endogenous cyanide production upon ingestion. Identifying cassava varieties with low cyanogenic potential is essential to improve safety and reduce the risk of toxicity.

Overall, the findings of this study indicate that cassava by-products were more frequently associated with variable effects on broiler performance, with negative responses occurring more frequently at dietary inclusion levels above 25%. However, the considerable heterogeneity among studies, differences in processing methods and dietary inclusion levels, and the predominance of studies with methodological limitations highlight the need for cautious interpretation.

In this context, the effective use of cassava by-products in poultry nutrition depends on appropriate processing, careful formulation, and appropriate dietary inclusion levels. Future research should focus on standardized experimental designs, comprehensive reporting of nutritional and toxicological parameters, and consistent reporting of measures of variability (e.g., SD and SEM) to better define safe and optimal inclusion strategies.

## Conclusion

Cassava by-products may represent alternative feed ingredients in broiler diets. However, they were more frequently associated with reduced growth performance at dietary inclusion levels above 25%, although responses varied according to the type of cassava by-product, processing method, and experimental conditions. Their effective use depends on appropriate processing, careful dietary formulation, and the adoption of suitable dietary inclusion levels to minimize nutritional limitations and maximize their potential as a partial substitute for corn. Future studies should adopt standardized experimental protocols and report measures of variability (e.g., SD and SEM) to strengthen the available evidence and better define safe inclusion levels for each cassava by-product.

## Data Availability

The data presented in this study will be made available in supplementary materials in Excel (.xslx) format.
